# Gastroprotective Activities of Ethanol Extract of Black Rice Bran (*Oryza sativa* L.) in Rats

**DOI:** 10.3390/molecules26133812

**Published:** 2021-06-22

**Authors:** Peerachit Tonchaiyaphum, Warangkana Arpornchayanon, Parirat Khonsung, Natthakarn Chiranthanut, Pornsiri Pitchakarn, Puongtip Kunanusorn

**Affiliations:** 1 Department of Pharmacology, Faculty of Medicine, Chiang Mai University, Chiang Mai 50200, Thailand; tpeerachit@gmail.com (P.T.); warangkana.arpornc@elearning.cmu.ac.th (W.A.); wparirat@yahoo.com (P.K.); cnatthak@gmail.com (N.C.); 2 Department of Biochemistry, Faculty of Medicine, Chiang Mai University, Chiang Mai 50200, Thailand; pornsiri.p@cmu.ac.th

**Keywords:** *Oryza sativa* L., black rice bran, antiulcer, gastroprotective, rats

## Abstract

Black rice is a type of rice in the *Oryza sativa* L. species. There are numerous reports regarding the pharmacological actions of black rice bran, but scientific evidence on its gastroprotection is limited. This study aimed to evaluate the gastroprotective activities of black rice bran ethanol extract (BRB) from the Thai black rice variety Hom Nil (*O. sativa* L. *indica*) as well as its mechanisms of action, acute oral toxicity in rats, and phytochemical screening. Rat models of gastric ulcers induced by acidified ethanol, indomethacin, and restraint water immersion stress were used. After pretreatment with 200, 400, and 800 mg/kg of BRB in test groups, BRB at 800 mg/kg significantly inhibited the formation of gastric ulcers in all gastric ulcer models, and this inhibition seemed to be dose dependent in an indomethacin-induced gastric ulcer model. BRB could not normalize the amount of gastric wall mucus, reduce gastric volume and total acidity, or increase gastric pH. Although BRB could not increase NO levels in gastric tissue, the tissue MDA levels could be normalized with DPPH radical scavenging activity. These results confirm the gastroprotective activities of BRB with a possible mechanism of action via antioxidant activity. The major phytochemical components of BRB comprise carotenoid derivatives with the presence of phenolic compounds. These components may be responsible for the gastroprotective activities of BRB. The 2000 mg/kg dose of oral BRB showed no acute toxicity in rats and confirmed, in part, the safe uses of BRB.

## 1. Introduction

Peptic ulcer disease (PUD) is one of the most common gastrointestinal diseases worldwide. PUD is classified as gastric ulcer and duodenal ulcer. The etiology of PUD is an imbalance between the defensive factors of gastroduodenal mucosa (e.g., the mucus–bicarbonate barrier, prostaglandin (PG), and mucosal blood flow) and the aggressive factors (such as hydrochloric acid and pepsin secretion, alcohol, psychological stress, and *Helicobacter pylori*). The increased aggressive factors cause erosive damage on gastroduodenal mucosa, while the compromised defensive factors expose the inner epithelial layer to acidity, leading to gastric ulcer formation. 

Initial therapy of PUD usually targets the management of underlying causes. It is confirmed that eradication of *H. pylori* helps in improving the healing rates of gastroduodenal ulcers and lowering the recurrent rates [1]. To date, proton pump inhibitors (PPIs) are the first-line antisecretory drug for PUD. Their mechanism of action is to irreversibly inhibit H^+^/K^+^-ATPase. However, PPIs are associated with many significant adverse effects, such as hypomagnesemia, cutaneous lupus erythematosus, osteoporosis-related fracture, acute kidney injury, increased risk of gastrointestinal infections, and *Clostridium difficile*-associated diarrhea. PPIs are not recommended for long-term use because of their possible adverse effects. For example, long-term use of omeprazole may cause methyl cobalamin deficiency. Other treatment options of PUD include acid neutralizing/inhibitory agents (such as antacids and histamine H_2_-receptor antagonists) and mucosal protective agents (such as PG analogs, sucralfate, colloidal bismuth compounds, and carbenoxolone) [2]. Histamine H_2_-receptor antagonists reduce gastric acid secretion via competitive inhibition at H_2_ receptor sites. Ranitidine has been removed from the market in many countries, because the level of the animal carcinogen N-nitrosodimethylamine was deemed unacceptable. Cimetidine is a potent inhibitor of cytochrome P450 enzymes, thereby causing interactions with several drugs [3]. Prostaglandin E_1_ (PGE_1_) analogues, e.g., misoprostol, help in increasing the number of defensive factors by stimulating bicarbonate and mucus secretion as well as in decreasing the amount of gastric acid by binding to PGE receptor 3 on parietal cells. Adverse effects of PGE_1_ analogues are diarrhea, abdominal pain, flatulence, nausea, and vomiting. The patients who are intolerant to these side effects often seek complementary and alternative medicines.

Nowadays, the use of medicinal plants with antiulcer effects has become more popular, especially in the countries in which these plants are common foods. Black rice is a type (ranked by color) of rice in the *Oryza sativa* L. species of the Poaceae family, which is mainly cultivated in Asia, including in the northern region of Thailand. Hom Nil is one of diverse varieties of Thai black rice in the *O. sativa* L. *indica* subspecies. Black rice has been used in traditional Chinese medicine for centuries to treat anemia and improve circulatory blood flow, renal function, and eye vision [4]. The bran of black rice has been shown to possess many pharmacological properties, including anti-inflammation [5], lowering of blood sugar level in mice, prevention of osteoporosis and Alzheimer’s disease [6,7], suppression of breast cancer cell metastasis [8], and promotion of the innate immune response [9,10]. From various extraction methods, it has been confirmed that the chemical components of black rice bran consist of phenolic acids, anthocyanins, γ-oryzanol, gallic acid, and α-tocopherol [11,12]. Alpha-tocopherol is well-known for its robust antioxidant activity [13]. In addition, it has been reported that α-tocopherol has a gastroprotective effect, as it promoted the healing of gastric ulcer in an acetic acid-induced ulcer model. Thus, this study aimed to evaluate the gastroprotective activities of black rice bran ethanol extract (BRB) from the Thai black rice variety Hom Nil (*O. sativa* L. *indica*) in animal models, its possible mechanisms of action, and its acute oral toxicity in rats.

## 2. Results

### 2.1. Phytochemical Screening of BRB Using Liquid Chromatography–Mass Spectrometry (LC-MS) and High-Performance Liquid Chromatography (HPLC) Analyses 

**Figure 1:**
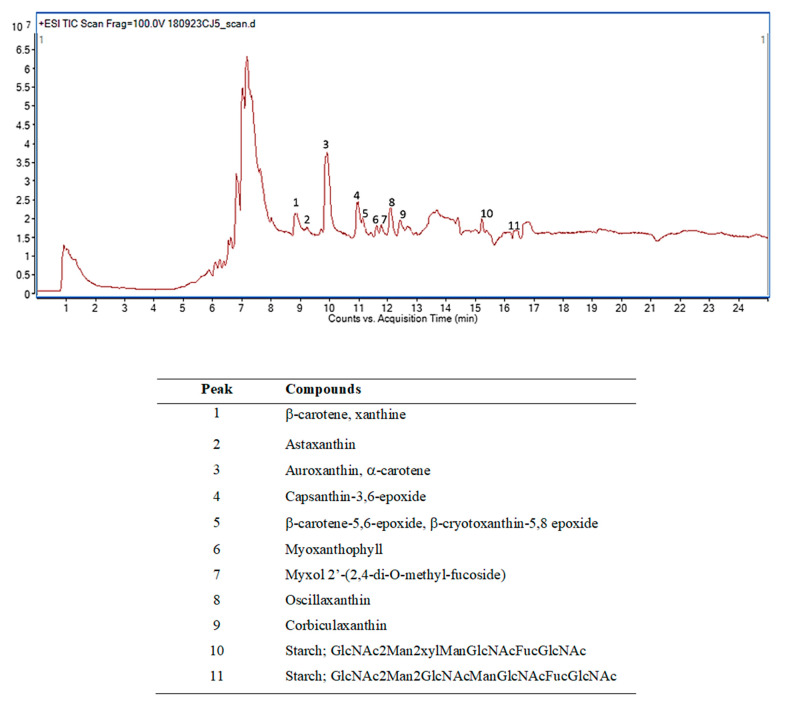
LC-MS chromatogram of BRB ethanol extract and the identified chemical constituents.

The phytochemical fingerprint of BRB is shown in Figure 1. Eleven compounds were identified from the chromatogram. In addition to starches, the major components comprised carotenoids, such as α-carotene and β-carotene, auroxantin, capxanthin, oscillaxantin, corbiculaxanthin, and astaxanthin. The presence of luteolin, apigenin, and vanillic acid was observed via HPLC analysis.

### 2.2. Gastroprotective Activities of BRB in Rats

#### 2.2.1. Effect of BRB on Acidified Ethanol (EtOH/HCl)-Induced Gastric Ulcer

**Figure 2:**
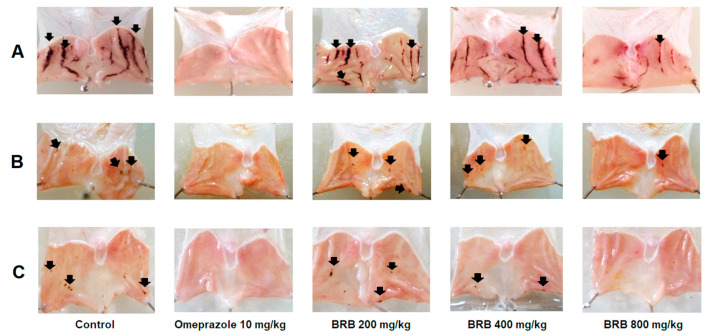
Comparison of the effects of pretreated omeprazole at 10 mg/kg and BRB at 200, 400, and 800 mg/kg on gastric surface from 3 different gastric ulcer models in rats versus control: (**A**) acidified ethanol-induced gastric ulcer; (**B**) indomethacin-induced gastric ulcer; (**C**) restraint water immersion stress-induced gastric ulcer. Black arrows indicate the characteristic necrotic bands forming gastric ulcers or the spots and small erosions forming gastric ulcers.

After oral administration of EtOH/HCl, severe gastric mucosal damage with multiple hemorrhagic spots was observed along the glandular segment of the stomach in the control rats (Figure 2A). The ulcer index of the control group was 179.20 ± 19.15 (Table 1). Pretreatment with 10 mg/kg of omeprazole showed the greatest inhibition of gastric ulcer formation of 85.34%, followed by 66.00% for BRB at 800 mg/kg and 48.77% for BRB at 400 mg/kg (*p* < 0.05).

#### 2.2.2. Effect of BRB on Indomethacin-Induced Gastric Ulcer

Damage of gastric glandular mucosa caused by indomethacin was visible in the control group with an ulcer index of 9.80 ± 3.84 mm (Figure 2B). On the contrary, the rats that received pretreatment with either omeprazole or BRB at all doses displayed significantly fewer gastric lesions. The maximum ulcer inhibition of BRB (78.85%) was seen at the dose of 800 mg/kg, while the inhibition rate of omeprazole was 85.20% (Table 2).

#### 2.2.3. Effect of BRB on Restraint Water Immersion Stress-Induced Gastric Ulcer

In the control group, spots of gastric ulcers were observed throughout the glandular portion of the stomach with an ulcer index of 6.63 ± 1.25 mm. The rats that were pretreated with omeprazole and 800 mg/kg of BRB showed a significant reduction in gastric ulcer formation (Figure 2C). The inhibition rates of omeprazole and the high dose BRB were 91.45% and 61.54%, respectively. BRB at the doses of 200 and 400 mg/kg failed to decrease the gastric ulcer caused by stress induction (Table 3).

### 2.3. Mechanisms of Gastroprotection of BRB

#### 2.3.1. Effect of BRB on Gastric Secretion Following Pyloric Ligation in Rats

After ligation of pylorus, the gastric volume and total acidity of the BRB group were slightly decreased, whereas the gastric pH was slightly increased. However, these findings are insignificant when compared to those of the control group (Table 4). Only the omeprazole-pretreated group showed significant elevation of gastric pH and reduction in gastric volume and total acidity.

#### 2.3.2. Effect of BRB on Gastric Wall Mucus Production in Rats

As compared with the normal control group, induction of gastric ulcer by acidified ethanol reduced the amount of gastric wall mucus in the ulcer control group (Table 5). The amount was as low as 6.78 ± 0.93 µg of alcian blue/g of wet stomach. Pretreatment with misoprostol before ethanol exposure significantly increased the production of gastric wall mucus to 9.73 ± 0.81 µg of alcian blue/g of wet stomach. Although 800 mg/kg of BRB in both BRB-normal and BRB-ulcer groups insignificantly elevated the amount of gastric wall mucus, under normal and ulcer conditions, when compared to the control groups, BRB could not normalize the amount of gastric wall mucus, and its effect was incomparable to that of misoprostol.

#### 2.3.3. Effect of BRB on the Levels of MDA and Nitric Oxide (NO) in Gastric Tissue

With acidified ethanol induction, the MDA level was very high in the ulcer control group (Table 6). However, the levels of MDA in gastric tissue were significantly low in the omeprazole and BRB groups. These levels also did not differ from those in the normal control group. Omeprazole at the dose of 10 mg/kg showed a significantly high level of NO in gastric tissue, similarly to that in the normal control group (Table 7). There was no significant difference in NO levels between gastric tissue derived from the rats that were pretreated with 800 mg/kg of BRB and that from the ulcer control rats.

#### 2.3.4. Effect of BRB on Antioxidant Activity Examined via 1,1-Diphenyl-2-picrylhydrazyl (DPPH) Assay and Total Phenolic Content

The relationship between DPPH and gallic acid is shown in Figure S1 (Supplementary Materials). The DPPH radical scavenging activities of BRB and gallic acid were present in dose-dependent manner. The potency of BRB was lower than that of gallic acid. The IC_50_ concentration of the DPPH radical scavenging activity of BRB was 151.84 ± 12.53 µg/mL, while that of gallic acid was 1.93 ± 0.86 µg/mL. The total phenolic content of BRB in the GAE value was 106.16 ± 8.43 mg gallic acid/g sample.

### 2.4. Acute Oral Toxicity Testing of BRB

Upon daily observation, animals did not exhibit any signs or symptoms of acute toxicities from the 2000 mg/kg of BRB via intragastric gavage. No mortality was reported within 24 h and until sacrifice. The changes in the rats’ body weight before and after 14 days of BRB administration are shown in Table 8. There was no change in the animals’ behaviors during this toxicity study. Necropsy showed no macroscopic changes in the internal organs. The weight of the organs of rats did not significantly differ between the high-dose BRB group and the control groups (Table 9).

## 3. Discussion

The gastroprotective effects of BRB from the Thai black rice variety Hom Nil (*O. sativa* L. *indica*) in the northern region of Thailand were confirmed by three models of gastric ulcers induced by acidified ethanol, indomethacin, and restraint water immersion stress in rats. BRB at 800 mg/kg significantly inhibited the formation of gastric ulcers in all gastric ulcer models. The inhibition of gastric ulcer formation appeared to be dose dependent in the indomethacin-induced gastric ulcer model. The efficacy of BRB at 800 mg/kg was almost comparable to that of omeprazole at 10 mg/kg (ulcer inhibition rate of BRB:omeprazole was 78.9%:85.2%). The BRB was able to insignificantly increase the gastric mucus production under normal and ulcer conditions, but its efficacy was still inferior to that of misoprostol. The DPPH assay demonstrated the radical scavenging activity of BRB. It is possible that the antioxidant property of BRB was also derived dose-dependently since BRB at a high dose (800 mg/kg) was also found to significantly reduce MDA lipid peroxidation and offered greater inhibition rates of gastric ulcer formation than those of the lower doses. However, the gastric volume, gastric pH, and total acidity were not significantly affected by BRB pretreatment in the pyloric ligation model. In addition, the gastroprotective activity of BRB was irrelevant to the endogenous NO.

Indomethacin is a classic type of NSAID that non-selectively inhibits cyclooxygenases (COX). Inhibition of COX-1 reduces PGs synthesis, thereby impairing the gastric mucosal defense. Indomethacin can also directly damage the mucosa by uncoupling the mitochondrial oxidative phosphorylation, resulting in overproduction of reactive oxygen species (ROS) [14]. During the restraint and immersion in water, the hypothalamic–pituitary–adrenal axis of the animals is stimulated, leading to an increased release of the adrenocorticotropic hormone that causes elevation of glucocorticoids in systemic circulation [15]. As a result, PG synthesis is inhibited, and the cytoprotective effects of PGE_2_-mediated prostaglandin E receptors, including an increase in mucus and bicarbonate secretion, a decrease in gastric acid secretion, and an improvement in mucosal blood flow, are impaired. Additionally, adrenaline from the sympathetic nervous system is associated with a release of gastrin [16]. Hence, the gastroprotective activities of BRB against stress in this study could be linked to antigastric secretion and the increase in PGs synthesis.

Nevertheless, the antigastric mechanism of BRB is still questionable. BRB pretreatment in the ligated pylorus could not increase gastric pH, reduce gastric secretion, or total acidity. These findings suggest that the mechanism of action of BRB is not related to antigastric secretion controlled by vagal stimulation. The results also indicate that the gastroprotective activity of BRB is irrelevant to the endogenous NO, which controls gastric mucosal blood flow. Similar to the findings of Trinovita et al., the extract of another color and variety of rice bran in *O. sativa* L. showed effective gastroprotection in the acidified ethanol-induced gastric ulcer model [17]. Interestingly, it was demonstrated that the 400 mg/kg of this color and variety of rice bran in the *O. sativa* L. extract provided a 66.8% gastric ulcer inhibition rate. The results regarding gastric acidity and production of gastric mucus are similar to those of omeprazole when the animals were pretreated for 7 days before exposure to ethanol [17]. However, in contrast to the method of Trinovita et al., the pretreatment of BRB in this study was given only 1 h before acidified ethanol exposure. The differences in color and variety of *O. sativa* L. rice bran, pretreatment duration, and extraction methods could affect the potency of different colors and varieties of *O. sativa* L. rice bran in protecting the gastric wall from pepsin and other exogenous irritants. A longer duration of pretreatment of BRB may increase its gastroprotective effect.

Oral administration of acidified ethanol is known to induce intracellular oxidative stress in gastric mucosa as well as disrupting gastric mucus, which leads to the perturbation of superficial epithelial mucosa, cellular necrosis, and gastric ulcer [18,19]. In this study, BRB was shown to reduce oxidative stress in the EtOH/HCl-induced gastric ulcer model as well as in the stress-induced gastric ulcer. The antioxidant activity of BRB was confirmed by the DPPH assay (the IC_50_ of BRB at 800 mg/kg was 151.84 ± 12.53 µg/mL, and the total phenolic assay was 106.16 ± 8.43 mg gallic acid/g sample). BRB demonstrated the inhibition of oxidative damage from the free radical scavenging of DPPH. The level of MDA in the gastric tissue of the BRB group was also significantly lower than that of the control animals following gastric ulcer induction by EtOH/HCl. In general, MDA is produced by ROS reactivity to the polyunsaturated fatty acid in the phospholipid membrane; therefore, MDA is often used as an indicator of oxidative stress in damaged tissues. A high level of MDA in gastric tissue typically indicates a high degree of lipid peroxidation and damage of gastric mucosa. Post BRB pretreatment, a lower level of MDA in the gastric tissue in the pretreated animals than that in the control animals suggested successful gastroprotection against oxidative stress.

Antioxidant activities and phytochemical constituents differ greatly among the different black rice varieties [11]. Black rice bran has higher contents of phenolics, flavonoids, and anthocyanins than those of white rice bran. The antioxidant activities of black rice bran are also greater. The bran of black rice contains numerous noble chemical components. The most important substances are γ-oryzanol, α-tocopherol, phenolic acids, anthocyanins, and gallic acid. It is believed that γ-oryzanol and tocopherol play the most crucial roles in the promotion and acceleration of gastric ulcer healing because of their potent antioxidant activities [13,17]. In this study, the LC-MS phytochemical screening of BRB also found high levels of carotenoid derivatives, for example, β-carotene, α-carotene, astaxanthin, auroxantin, and capsanthin. Similar to other plants containing high carotenoid derivatives, it is possible that astaxanthin and β-carotene in BRB are the additional factors that provide gastroprotective and antioxidant properties [20,21]. According to Murata et al., astaxanthin remarkably decreases the severity of mucosal damage in the ethanol and aspirin-induced gastric ulcers in murine models in the same way observed in this study [21]. Moreover, in this study, HPLC analysis revealed the phenolic compounds, such as vanillic acid, luteolin, and apigenin, in BRB. Vanillic acid has been reported to exert an inhibitory effect on the inflammatory response via the regulation of NF-κB p65 activation in dextran sulfate sodium-induced ulcerative colitis [22]. Luteolin is well known for its antiulcer activity in the reserpine-induced gastric ulcer in mice [23]. Furthermore, apigenin is well known as a natural product that can be used in alternative therapy against inflammation-related chronic diseases, including insulin resistance, diabetes mellitus, fatty liver, colitis, and gastric ulcer [24]. These phenolic compounds therefore may also be responsible for a mechanism of the antiulcer effect of BRB.

In each plant, bioactive compounds and their various solubility properties in different solvents are diverse. Therefore, a suitable extraction solvent for individual plant materials is required. Asian populations, including Chinese, Vietnamese, and Thai populations, widely use herb plants as folk medicine. These herbs are soaked for at least 3–5 days to produce medicinal liquors. Thus, the extraction protocol in this study mimics the instructions for the use of the plant sample in daily life. Furthermore, ethanolic extraction is less toxic than extraction using methanol and hydrochloric acid when extracting anthocyanins and other phenolic compounds, which are well known as the bioactive components of black rice [25,26].

Furthermore, there was no sign of acute toxicity in the 2000 mg/kg of BRB. Neither treatment-related toxicities nor mortality was observed during the 14 days of study. The LD_50_ of BRB was estimated to be greater than 2000 mg/kg. According to the Globally Harmonized Classification System (GHS) for chemical substances and mixtures, any substance with LD_50_ of more than 2000 to 5000 mg/kg is categorized as unclassified or category 5. This result provides one important data on the non-toxicity profile of acute, high-dose BRB. However, the adverse effects of long-term BRB consumption are unknown, because chronic toxicity testing has not yet been conducted. Further study is warranted before clinical application.

## 4. Materials and Methods

### 4.1. Plant Collection and Preparation of Ethanol Extract

Black rice bran from the Thai black rice variety Hom Nil (*O. sativa* L. *indica*) was bought from Lanna Rice Research Center, Chiang Mai University, Chiang Mai, Thailand. Black rice grains were dehusked and polished using a rice dehusker and a rice milling machine, which allowed for approximately 7.20% (*w*/*w*) of the milled rice bran to be obtained. The bran was sieved through a 60-mesh strainer, stored at room temperature, and then macerated in 80% ethanol at a ratio of 1:10 (g:L) for 24 h. Later, the maceration of BRB was filtrated and evaporated under reduced pressure using a vacuum rotary evaporator followed by lyophilization. The product was stored at −20 °C. The final yield of black rick bran in 80% ethanol extract per dry weight was 10.84%.

### 4.2. Phytochemical Screening

#### 4.2.1. LC-MS Analysis

The phytochemical fingerprint of BRB was obtained by semi-quantitative LC-MS analysis. The BRB was dissolved in methanol (HPLC grade) and filtered with 0.22 µm nylon filter. Chromatographic separations were performed using a mobile phase composed of 0.1% formic acid in water (A) and 0.1% formic acid in acetonitrile (B). The optimized gradient program started with 5% of B followed by a gradient of up to 50% for 6 min, 80% for 12 min, and 95% for 15 min with a flow rate of 0.3 mL/min, and the injection volume was 5 mL. An Eclipse plus C18 column (2.1 × 100 mm 1.8 µm) was used, and the column temperature was maintained at 40 °C. The working parameters of the mass spectrometer were as follows: desolvation line temperature, 320 °C; ion source voltage, 3500 V; nebulizing gas flow, 9 L/min; nebulizer, 45 psi; sheath gas heater, 350 °C; sheath gas, flow 11 L/min. The chemical constituents of the extract were identified by comparing the relative retention times and mass spectra with the data in the W8N08.L database (John Wiley and Sons, Inc., New York, NY, USA).

#### 4.2.2. HPLC Analysis

The phenolic compounds of BRB were screened using HPLC analysis. The chromatograms were obtained from UV absorbance at 250 and 330 nm. The Purospher^®^ RP-18 Endcapped HPLC Columns with a temperature of 25 °C were used. The mobile phase comprised 0.1% formic acid in water and a methanol flow rate of 0.7 mL/min. The injection volume was 10 mL. Spectra for all peaks were obtained between 200 and 400 nm. Gradient solutions were 5/90, 10/80, 15/70, 20/50, 25/30, 30/10, and 40/90 min/%A.

### 4.3. Experimental Animals

Male Sprague–Dawley rats weighting between 200 and 250 g were purchased from the National Laboratory Animal Center, Mahidol University, Salaya, Nakorn Pathom, Thailand. All animals were acclimatized in an animal room and maintained at 24 ± 1 °C and a 12 h light–12 h dark cycle for at least 1 week before starting the experiment. The rats had free access to water and standard diets (082 C.P. MICE FEED, S.W.T. Co., Ltd., Samut Prakan, Thailand). The number of animals used in each animal study was in accordance with that in each standard model or guideline. This study was approved by the Animal Ethics Committee of the Faculty of Medicine, Chiang Mai University, Chiang Mai, Thailand (Protocol Number 06/2560).

### 4.4. Evaluation of Gastroprotective Activities

#### 4.4.1. Acidified Ethanol (EtOH/HCl)-Induced Gastric Ulcer

A modified method of the Mizui and Doteuchi experiment was performed [27]. The rats were starved for 48 h but allowed free access to water. Randomization was performed to divide the rats into 5 groups (*n* = 6). The control group received 5 mL/kg of 5% Tween 80 via intragastric gavage, while the reference group received 10 mg/kg of omeprazole. The test groups received 200, 400, and 800 mg/kg of BRB. One hour later, all rats were administered (by gavage) with 1.0 mL EtOH/HCl (60 mL EtOH, 1.7 mL HCl, 38.3 mL distilled water) to induce gastric ulcer and then euthanized 1 h later. Incision was made along the greater curvature to open and remove the stomach. The quantification of gastric lesions of each stomach was performed under a binocular magnifier. Then, the ulcer index and the percentage of gastric ulcer inhibition were calculated. The measurement of each gastric lesion was performed along its longest diameter, and five petechiae were considered equivalent to a 1 mm length of lesion. The mean ulcer index of each group was then calculated (the sum of the ulcer lengths divided by the number of rats in that group) [28].

#### 4.4.2. Indomethacin-Induced Gastric Ulcer

Some adaptations were applied to the method described by Nwafor et al. [29]. Most steps were similar to the EtOH/HCl-induced gastric ulcer model, with the exception of the substance used to induce gastric ulcer; 100 mg/kg of indomethacin in 0.5% carboxymethylcellulose was administered via intragastric gavage. After 5 h, the rats in all groups were euthanized and measured for gastric ulcer.

#### 4.4.3. Restraint Water Immersion Stress-Induced Gastric Ulcer

The experiment followed the method of Takagi et al. with slight modification [30]. To induce gastric ulcer, the rats were individually placed in stainless steel cages, restrained, and immersed in a cold water bath (22 ± 2 °C) up to the level of xiphoid. Intragastric gavage of 5% Tween 80, omeprazole (10 mg/kg), or BRB (200, 400 or 800 mg/kg) was administered 1 h earlier. Five hours later, the animals were sacrificed. Determination of gastric ulcer from the excised stomach was carried out.

### 4.5. Investigation of Gastroprotective Mechanisms

#### 4.5.1. Pylorus Ligation

After 48 h of fasting, the rats were randomly allocated into 3 groups (*n* = 6 each). The control group received 5 mL/kg of 5% Tween 80 via intragastric gavage, while the reference group received 10 mg/kg of omeprazole. The test group received BRB at a dose of 800 mg/kg. One hour later, pyloric ligation was performed [31]. The gastric content was collected from all 18 rats and centrifuged to measure the volume, pH, and total acidity. Titration with 0.1 N sodium hydroxide was performed using phenolphthalein.

#### 4.5.2. Quantification of Gastric Wall Mucus

The rats were randomly allocated into 5 groups (*n* = 6 each). All of them were fasted for 48 h but had free access to water. No ulcer induction was made to the animals in the “normal control group” (received 5 mL/kg of 5% Tween 80 via intragastric gavage) and the animals in the “BRB-normal group” (received BRB at 800 mg/kg). Gastric ulcers were induced using acidified ethanol in the “ulcer control group” (received 5 mL/kg of 5% Tween 80), “misoprostol group” (received 100 µg/kg of misoprostol), and “BRB-ulcer group” (received BRB at 800 mg/kg). One hour after gastric ulcer induction, stomach excision was performed under euthanasia. Alcian blue was used to quantify the gastric wall mucus [32]. The glandular part of each stomach was weighed before staining with 0.1% (*w*/*v*) alcian blue (10 mL). Sucrose solution (0.25 M) was used to rinse the excess dye from the stomach. The dye–gastric mucous complex was extracted by immersion in 0.5 M magnesium chloride (10 mL) for 2 h. The same amounts of the aqueous alcian blue extract and ether were mixed together. The aqueous layer was separated after centrifugation at 4120× *g* for 10 min, and its absorbance was measured at 580 nm. The quantity of alcian blue extract was calculated. The amount of gastric visible mucus was recorded as µg of alcian blue/g of wet stomach.

#### 4.5.3. Determination of MDA as Lipid Peroxidation Product

The four groups of rats were used to determine the tissue levels of MDA in gastric mucosa. The “normal control group” received 5 mL/kg of 5% Tween 80 without gastric ulcer induction. The other three groups with gastric ulcer induced by acidified ethanol included the “ulcer control group” (received 5 mL/kg of 5% Tween 80), the “omeprazole group” (received 10 mg/kg of omeprazole), and the “BRB-ulcer group” (received BRB at 800 mg/kg). The removed stomach was measured, and 0.2 mL of the tissue homogenate supernatant was used. Then, 0.2 mL of 8.1% sodium dodecyl sulfate, 1.5 mL of 20% acetic acid solution, and 1.5 mL of 0.8% aqueous solution thiobarbituric acid were mixed with the sample. The mixture was then diluted with distilled water to 4.0 mL and heated at 95 °C for 60 min. After cooling, 1.0 mL of distilled water and 5.0 mL of the mixture of n-butanol and pyridine (15:1, *v*/*v*) were added and shaken vigorously. After centrifugation at 4000 rpm for 10 min, the organic layer was transferred, and the absorbance was measured at 532 nm by a UV–visible spectrophotometer. The standard curves were acquired using 0–20 mM of MDA.

#### 4.5.4. Determination of NO

The four groups of rats, similar to in the determination of MDA, were used to determine the levels of NO in gastric tissue. Samples were prepared by homogenizing the excised gastric tissue in a chilled phosphate buffer, which was centrifuged at 3500 rpm for 10 min at 4 °C. Then, 100 µL of the clear supernatant was kept at −80 °C, mixed with Griess reagent, and incubated at room temperature for 10 min. A spectrophotometer at 540 nm was used to determine NO content.

#### 4.5.5. Determination of Antioxidant Activity by DPPH Assay

An amount of 0.5 mL of BRB was added to 0.5 mL of absolute ethanol at different concentrations and then mixed with 0.6 mL of 100 mM DPPH for 30 min under light protection. The absorbance was read at 517 nm on a spectrophotometer. The DPPH radical scavenging activity was calculated. The antioxidant activity was expressed as gallic acid equivalent (GAE).

#### 4.5.6. Determination of Total Phenolic Contents

A modification of the Folin–Ciocalteu method was performed. BRB solution at different concentrations (0.2 mL) was mixed with 10% Folin–Ciocalteu solution (1.0 mL) plus 7.5% Na_2_CO_3_ solution (0.8 mL). A spectrophotometer at 765 nm was used to determine the total phenolic compounds (expressed as GAE).

### 4.6. Acute Oral Toxicity Test

The acute oral toxicity test was performed following the internationally accepted OECD Test Guideline 420 [33]. Ten adult female Sprague–Dawley rats weighing between 180 and 200 g were randomly divided into 2 groups. Animals in the control group received 5% Tween 80 at 5 mL/kg via oral gavage, while animals in the test group received a high dose of BRB (2000 mg/kg). General appearances and behavioral signs were closely monitored. Changes in skin, fur, eyes, and mucous membrane were recorded at 1, 2, 4, and 6 h and every 24 h daily for 14 days. Sacrifices and necropsies were made on the 15th day to examine the gross pathological changes in the internal organs.

### 4.7. Statistical Analysis

All data are expressed as mean ± standard error of the mean (SEM). One-way analysis of variance (ANOVA) followed by the post hoc least-significant difference (LSD) test was used to determine the statistical significance (*p* < 0.05) of the differences among groups. Student’s *t*-test was used to determine the statistical significance (*p* < 0.05) from the control group.

## 5. Conclusions

Ethanol extract of black rice bran from the Thai black rice variety Hom Nil (*O. sativa* L. *indica*) at the dose of 800 mg/kg displayed effective gastroprotection against gastric ulcers in all gastric ulcer models, and this inhibition appeared to be dose dependent in the indomethacin-induced gastric ulcer model. BRB could not normalize the amount of gastric wall mucus, reduce gastric volume and total acidity, or increase gastric pH. Although BRB could not increase NO levels in gastric tissue, the tissue MDA levels could be normalized with DPPH radical scavenging activity. These results confirm the gastroprotective activities of BRB with a possible mechanism of action via antioxidant activity. The major phytochemical components of BRB comprise carotenoid derivatives with the presence of phenolic compounds. These components may be responsible for the gastroprotective activities of BRB. The 2000 mg/kg dose of oral BRB showed no acute toxicity in rats and confirmed, in part, the safe uses of BRB. These findings provide scientific data to support the potential of developing BRB as an alternative traditional medicine in the future. Further chronic toxicity study is warranted before clinical application.

## Figures and Tables

**Table 1:** Effect of BRB on gastric mucosa in the acidified ethanol-induced gastric ulcer.

Group	Dose (mg/kg)	Ulcer Index (mm)	Inhibition (%)
Control	-	179.20 ± 19.15	-
Omeprazole	10	26.27 ± 8.16 *	85.34
BRB	200	167.03 ± 23.39	6.78
	400	91.80 ± 8.92 *	48.77
	800	60.93 ± 10.70 *	66.00

Data are represented as mean ± SEM (*n* = 6). One-way analysis of variance (ANOVA) followed by the post hoc least-significant difference (LSD) test was used to determine the significant difference from the control group (5% Tween 80); * *p* < 0.05.

**Table 2:** Effect of BRB on gastric mucosa in the indomethacin-induced gastric ulcer.

Group	Dose (mg/kg)	Ulcer Index (mm)	Inhibition (%)
Control	-	9.80 ± 3.84	-
Omeprazole	10	1.45 ± 0.55 *	85.20
BRB	200	5.48 ± 1.27 *	44.05
	400	4.48 ± 0.77 *	54.25
	800	2.36 ± 0.37 *	78.85

Data are represented as mean ± SEM (*n* = 6). One-way analysis of variance (ANOVA) followed by the post hoc least-significant difference (LSD) test was used to determine the significant difference from the control group (5% Tween 80); * *p* < 0.05.

**Table 3:** Effect of BRB on gastric mucosa in the restraint water immersion stress-induced gastric ulcer.

Group	Dose (mg/kg)	Ulcer Index (mm)	Inhibition (%)
Control	-	6.63 ± 1.25	-
Omeprazole	10	0.57 ± 0.23 *	91.45
BRB	200	5.33 ± 0.93	19.56
	400	4.80 ± 1.08	27.60
	800	2.55 ± 0.50 *	61.54

Data are represented as mean ± SEM (*n* = 6). One-way analysis of variance (ANOVA) followed by the post hoc least-significant difference (LSD) test was used to determine the significant difference from the control group (5% Tween 80); * *p* < 0.05.

**Table 4:** Effect of BRB on gastric secretion after pyloric ligation.

Group	Gastric Volume (mL)	Gastric pH	Total Acidity (mEq/100 g/h)
Control	4.28 ± 0.36	2.57 ± 0.34	3.79 ± 1.47
Omeprazole (10 mg/kg)	2.65 ± 0.26 *	5.47 ± 0.19 *	0.05 ± 0.01 *
BRB (800 mg/kg)	3.83 ± 0.11	2.97 ± 0.53	2.99 ± 1.55

Data are represented as mean ± SEM (*n* = 6). One-way analysis of variance (ANOVA) followed by the post hoc least-significant difference (LSD) test was used to determine the significant difference from the control group (5% Tween 80); * *p* < 0.05.

**Table 5:** Effect of BRB on gastric mucus production with or without gastric ulcer induction by acidified ethanol.

Group	Dose	Amount of Gastric Wall Mucus (µg of Alcian Blue/g of Wet Stomach)
Normal control ^a^	-	8.80 ± 0.67
BRB-normal ^a^	800 mg/kg	9.49 ± 0.57 *
Ulcer control ^b^	-	6.78 ± 0.93 ^#^
Misoprostol ^b^	100 µg/kg	9.73 ± 0.81 *
BRB-ulcer ^b^	800 mg/kg	7.54 ± 0.92 ^#^

Data are represented as mean ± SEM (*n* = 6). ^a^ Without gastric ulcer induction. ^b^ With gastric ulcer induction by EtOH/HCl. One-way analysis of variance (ANOVA) followed by the post hoc least-significant difference (LSD) test was used to determine the significant difference between groups (* *p* < 0.05 compared with the ulcer control group; ^#^ *p* < 0.05 compared with the normal control group).

**Table 6:** Effect of BRB on MDA levels in gastric tissue as lipid peroxidation product with gastric ulcer induction by acidified ethanol.

Group	Dose (mg/kg)	Level of MDA in Gastric Tissue (µmol/g Protein)
Normal control ^a^	-	111.39 ± 9.60 *
Ulcer control ^b^	-	175.31 ± 6.68 ^#^
Omeprazole ^b^	10	93.63 ± 3.82 *
BRB-ulcer ^b^	800	105.28 ± 7.13 *

Data are represented as mean ± SEM (*n* = 6). ^a^ Without gastric ulcer induction. ^b^ With gastric ulcer induction by EtOH/HCl. One-way analysis of variance (ANOVA) followed by the post hoc least-significant difference (LSD) test was used to determine the significant difference between groups (* *p* < 0.05 compared with the ulcer control group; ^#^ *p* < 0.05 compared with the normal control group).

**Table 7:** Effect of BRB on NO levels in gastric tissue with gastric ulcer induction by acidified ethanol.

Group	Dose (mg/kg)	Level of NO in Gastric Tissue (µg/g of Protein)
Normal control ^a^	-	13.78 ± 0.36 *
Ulcer Control ^b^	-	6.19 ± 0.27
Omeprazole ^b^	10	11.50 ± 0.30 *
BRB-ulcer ^b^	800	5.46 ± 0.32

Data are represented as mean ± SEM (*n* = 6). ^a^ Without gastric ulcer induction. ^b^ With gastric ulcer induction by EtOH/HCl. One-way analysis of variance (ANOVA) followed by the post hoc least-significant difference (LSD) test was used to determine the significant difference from the ulcer control group; * *p* < 0.05.

**Table 8:** Effect of high-dose BRB on body weight of rats in acute oral toxicity testing.

Group	Body Weight (g)			Total Weight Changes (g)	Average Weight Changes (%)
	Day 1	Day 7	Day 14		
Control	174.0 ± 2.2	202.0 ± 1.8	202.0 ± 1.8	28.0 ± 3.4	16.09
BRB 2000 mg/kg	164.0 ± 2.2	188.0 ± 3.4	188.0 ± 1.8	34.0 ± 2.2	20.73

Data are represented as mean ± SEM (*n* = 5). Student’s *t*-test was used to determine the significant difference from the control group (5% Tween 80).

**Table 9:** Internal organ weights (g) of rats in the control and BRB groups after 14 days of acute toxicity testing.

Group	Heart	Liver	Spleen	Pancreas	Uterus	Lung	Kidney		Ovary	
							L	R	L	R
Control	0.86 (0.06)	5.54 (0.46)	0.55 (0.04)	0.48 (0.06)	0.33 (0.04)	1.80 (0.11)	0.67 (0.07)	0.72 (0.10)	0.08 (0.02)	0.08 (0.02)
BRB 2000 mg/kg	0.77 (0.02)	4.54 (0.14)	0.46 (0.02)	0.40 (0.05)	0.24 (0.04)	1.57 (0.08)	0.64 (0.03)	0.67 (0.02)	0.06 (0.02)	0.06 (0.02)

Data are represented as mean (SEM) (*n* = 5). Student’s *t*-test was used to determine the significant difference from the control group (5% Tween 80).

## Data Availability

The data presented in this study are available on request from the corresponding author.

## Supplementary Materials

The following is available online. Figure S1: The log concentrations and % DPPH radial scavenging activities of ethanol extract of BRB and gallic acid.

## Sample Availability

Samples of the compounds are not available from the authors.
