# Supplementary material for: Gastroprotective Activities of Ethanol Extract of Black Rice Bran (Oryza sativa L.) in Rats
Source: Molecules. 2021 Jun 22;26(13):3812. doi: 10.3390/molecules26133812 (PMC8270266; doi:10.3390/molecules26133812)
Supplement: Supplementary file 1 [file molecules-26-03812-s001.zip › molecules-1261808-supplementary.pdf]

Supplementary Materials

Gastroprotective Activities of Ethanol Extract of Black Rice Bran (*Oryza sativa* L.) in Rats

Peerachit Tonchaiyaphum [1], Warangkana Arpornchayanon [1], Parirat Khonsung [1], Natthakarn Chiranthanut [1], Pornsiri Pitchakarn [2] and Puongtip Kunanusorn [1,*]

1 Department of Pharmacology, Faculty of Medicine, Chiang Mai University, Chiang Mai 50200, Thailand; tpeerachit@gmail.com (P.T.); warangkana.arpornc@elearning.cmu.ac.th (W.A.); wparirat@yahoo.com (P.K.); cnatthak@gmail.com (N.C.)

2 Department of Biochemistry, Faculty of Medicine, Chiang Mai University, Chiang Mai 50200, Thailand; pornsiri.p@cmu.ac.th

* Correspondence: puongtip.k@cmu.ac.th; Tel.: +66-53-935-353

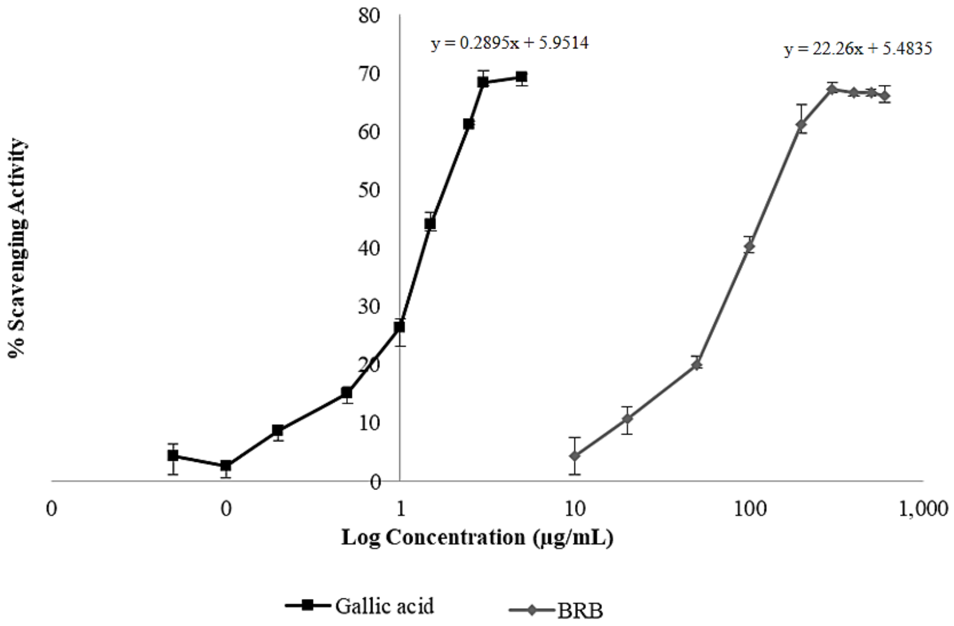

Figure S1. The log concentrations and % DPPH radial scavenging activities of ethanol extract of BRB and gallic acid.
